# Importance of Recognizing Renal Tubular Disorders as a Cause of Bone Hypomineralization and Fractures in Adults

**DOI:** 10.3390/diagnostics16121898

**Published:** 2026-06-18

**Authors:** Carlos Perez Gomes, Alinie da Silva Pichone, Maria Lucia Fleiuss de Farias

**Affiliations:** 1 Nephrology Division, Federal University of Rio de Janeiro (UFRJ), Rio de Janeiro 21941-617, Brazil; al_pichone@yahoo.com; 2 School of Medicine and Surgery, Federal University of the State of Rio de Janeiro (UNIRIO), Rio de Janeiro 20270-330, Brazil; 3 Endocrinology Division, Faculty of Medicine, Federal University of Rio de Janeiro (UFRJ), Rio de Janeiro 21941-617, Brazil; fleiuss@hucff.ufrj.br

**Keywords:** osteoporosis, renal tubulopathy, fractures, hypercalciuria, hypophosphatemia, acidosis

## Abstract

Renal tubular disorders are often overlooked causes of acquired or inherited bone hypomineralization and fragility fractures in adults. The proximal tubule reabsorbs glucose, phosphate, low-molecular-weight proteins, amino acids, bicarbonate, and much of the sodium, potassium, chloride, and calcium. The distal nephron—the thick ascending limb of the loop of Henle, the distal convoluted tubule, and the collecting duct—regulates urine concentration and dilution, maintains acid-base balance via urinary proton secretion, and controls electrolytes, including sodium, potassium, magnesium, and calcium. Tubular defects may cause hyperphosphaturia (high urinary phosphate), hypercalciuria (high urinary calcium), or chronic metabolic acidosis (renal tubular acidosis, RTA). These changes weaken bone mineralization, disrupt bone turnover, and raise the risk of muscle weakness and fractures. This review summarizes acquired and genetic tubulopathies linked to hyperphosphaturia, hypercalciuria, and RTA and outlines a practical diagnostic approach for outpatients with bone fragility and suspected renal tubulopathy.

## 1. Introduction

Renal tubular disorders should be considered in the differential diagnosis of bone diseases, particularly given reports of increased incidence of secondary rickets due to renal tubulopathy, with a corresponding impact on mortality in recent years [1]. Their clinical manifestations can mimic those of more common bone conditions but often require different management. Bone strength depends on both bone mass and mineralization. Hypophosphatemia and renal phosphaturia directly impair mineralization, causing rickets or osteomalacia. Persistent urinary calcium loss (hypercalciuria), magnesium deficiency, or chronic metabolic acidosis can accelerate bone loss and skeletal fragility [2,3]. Tubular disorders may first present as bone pain, muscle weakness, or fractures. These conditions are sometimes misdiagnosed as ‘idiopathic osteoporosis’ if renal causes are not considered. Several case series and reviews have reported osteomalacia, recurrent low-impact fractures, and proximal muscle weakness in adults. For instance, these cases include patients with previously unrecognized Fanconi-type proximal tubulopathies. They also include drug-induced tubular dysfunction, notably from tenofovir, adefovir, and ifosfamide, as well as acquired or genetic calcium/phosphate-wasting disorders and renal tubular acidosis [4]. Early identification of these conditions permits precise interventions, such as discontinuing the causative drug, administering phosphorus or vitamin D supplementation, initiating alkali therapy, administering thiazide diuretics, and providing disease-specific treatments. These measures may help prevent further fractures [5].

## 2. Methods

We conducted a literature search for the present narrative review using PubMed, Cochrane, the National Library of Medicine, and Embase between October 2025 and March 2026. The search included the terms: “tubulopathy”, “renal tubular disorders”, “hyperphosphaturia”, “hypercalciuria”, “renal tubular acidosis”, “secondary osteoporosis”, “metabolic bone disease”, “bone fragility”, “bone mineralization”, “fractures and renal”, “fractures and tubulopathy”, “osteoporosis and renal”, and “bone and renal”.

First, we screened the articles based on their titles and abstracts. We considered studies published between 1995 and 2026 that focused on the diagnosis, prognosis, or treatment of bone mineral disorders associated with renal tubulopathies. Using the search terms described above individually, we initially retrieved 48,059 articles. After cross-referencing the data—primarily by linking hereditary or acquired tubulopathies to bone involvement—and prioritizing clinical trials, observational cohorts, case series, and review articles published in the last 5 years, we ultimately selected 82 articles.

## 3. How Does the Interaction Among Hyperphosphaturia, Hypercalciuria, and Renal Tubular Acidosis (RTA) Contribute to Complex Disruptions in Mineral Metabolism and Compromise Skeletal Integrity?

### 3.1. Hyperphosphaturia

Phosphate, an essential part of hydroxyapatite, is necessary for cellular metabolism. The kidneys maintain systemic phosphate homeostasis primarily by reabsorbing phosphate in the proximal tubule. In hyperphosphaturia, impaired phosphate reabsorption leads to persistent hypophosphatemia, which compromises bone mineralization and results in osteomalacia, pseudofractures, and increased bone fragility. Phosphate wasting results from downregulation of type II sodium-dependent phosphate cotransporters (NaPi-IIa, NaPi-IIc) at the apical membrane of proximal tubular cells, a process regulated by systemic factors [6]. Both parathyroid hormone (PTH) and fibroblast growth factor 23 (FGF23), along with its cofactor α-Klotho, decrease renal phosphate reabsorption. PTH and FGF23 downregulate NaPi cotransporters, whereas FGF23 also inhibits 1α-hydroxylase, reducing the synthesis of active vitamin D (1,25(OH)_2_D). Reduced levels of active vitamin D further limit intestinal absorption of calcium and phosphate. Disruptions at any of these regulatory points increase urinary phosphate excretion, and hypophosphatemia impairs bone health through multiple mechanisms [7]. First, defective matrix mineralization arises because phosphate is required for hydroxyapatite crystal formation in osteoid. Chronic hypophosphatemia reduces inorganic phosphate availability, leading to widened osteoid seams, decreased mineralization density, and clinical osteomalacia, characterized by bone pain, muscle weakness, pseudofractures, and fragility fractures. Osteomalacia is characterized by low bone turnover [8]. Second, FGF23 exerts direct skeletal and endocrine effects, including paracrine actions within bone, some of which are independent of α-Klotho, that modify osteocyte and osteoblast function and matrix mineralization. Elevated FGF23 is associated with poor bone mineralization and increased fragility, as demonstrated by histologic and imaging studies [8,9]. Third, FGF23-mediated suppression of 1α-hydroxylase reduces 1,25(OH)_2_D, thereby decreasing intestinal calcium and phosphate absorption. Lower vitamin D levels further worsen mineralization and may induce secondary hyperparathyroidism, which increases bone turnover and reduces bone mass, exacerbating fragility [10]. Finally, recent research describes extracellular matrix “stenciling,” in which local enzymes and inhibitors, such as alkaline phosphatase and pyrophosphate-generating enzymes, regulate mineral deposition. Hypophosphatemic conditions disturb this homeostasis, leading to focal defects in mineral nucleation and expansion. These microscopic alterations soften bone and increase fracture risk [11].

Chronic phosphate waste produces two overlapping skeletal phenotypes. Osteomalacia, characterized by inadequate mineralization of new osteoid, presents as wide unmineralized osteoid seams, pseudofractures, bone pain, and increased fragility. This phenotype is typical of sustained hypophosphatemia, as observed in genetic disorders such as X-linked hypophosphatemic rickets (XLH) and in acquired conditions such as tumor-induced osteomalacia (TIO) [11]. In contrast, vitamin D deficiency and secondary hyperparathyroidism increase bone turnover, reduce cortical thickness, and compromise bone microarchitecture. Although these changes differ from those of classical osteomalacia, they further increase fracture risk. Genetic alterations may additionally disrupt osteocyte-matrix interactions, leading to enthesopathies and impairing mechanical loading and skeletal integrity [12].

Multiple causes can decrease renal phosphate reabsorption or suppress active vitamin D synthesis. These include hormonal, genetic, acquired proximal tubular dysfunction, and paraneoplastic processes. All these mechanisms result in chronic hypophosphatemia, impaired bone mineralization (osteomalacia), altered bone microarchitecture, and a higher risk of fragility fractures [6]. Suppression of active vitamin D is significant in the setting of impaired proximal tubular function. Some genetic disorders cause tubular injury and phosphate loss, stimulating 1,25(OH)_2_D production. In other conditions, such as chronic kidney disease (CKD), active vitamin D levels are reduced. Both scenarios negatively affect bone mineralization. Certain genetic phosphate-wasting disorders, such as hypophosphatemic rickets with hypercalciuria (HHRH), are defined by high 1,25(OH)_2_D and hypercalciuria [5]. Other causes of renal phosphate waste involve FGF23. For example, excess circulating FGF23 from genetic upregulation—such as PHEX mutations in XLH, DMP1 or ENPP1 mutations in other hereditary hypophosphatemias—changes in NaPi cotransporters, or FGF23-secreting tumors in TIO can lead to renal phosphate wasting with skeletal consequences [13]. Advances in molecular genetics have refined the classification of FGF23-mediated and non-FGF23 hypophosphatemic disorders [12]. XLH and TIO are the principal hereditary disorders that link hyperphosphaturia to osteomalacia and fragility fractures [10,14]. Other causes include primary or tertiary hyperparathyroidism, in which chronic elevation of PTH leads to bone loss and disrupts mineral homeostasis in several ways [15]. Generalized proximal tubulopathies, such as Fanconi syndrome, are characterized by phosphaturia, aminoaciduria, glucosuria, and bicarbonaturia. Causes include inherited disorders and acquired forms from drugs (such as tenofovir or ifosfamide), heavy metals, and mitochondrial toxins [5]. Some malignancies or systemic illnesses can also indirectly affect phosphate handling [8].

Clinically, chronic hyperphosphaturia presents as bone pain, diffuse muscle weakness, delayed fracture healing, pseudofractures, rickets, and, in children, growth failure [16]. Laboratory findings include low serum phosphate, high urinary fractional excretion of phosphate (FEP > 5%), inappropriately normal or low 1,25(OH)_2_D, and often elevated FGF23. Bone markers, such as elevated alkaline phosphatase, and radiographs showing Looser zones or pseudofractures aid diagnosis. Dual-energy X-ray absorptiometry (DXA) may show low bone mineral density (BMD), but this can be misleading since osteomalacia may coexist with normal BMD. Differentiating FGF23-mediated causes (e.g., XLH or TIO) from non-FGF23-mediated causes (e.g., proximal tubulopathies or PTH-mediated disorders) is essential. This distinction guides subsequent tests, including genetic analysis and, when necessary, tumor localization using functional imaging [3].

### 3.2. Hypercalciuria

Hypercalciuria is a common metabolic problem in calcium kidney stone disease and can also occur without symptoms. Beyond its role in nephrolithiasis, hypercalciuria affects bone health. Studies show lower bone mineral density (BMD) and higher osteopenia and osteoporosis rates in people with hypercalciuria. Up to one-third of osteoporosis patients have hypercalciuria [17,18].

More than 60% of serum calcium filtered by the glomerulus is reabsorbed in the proximal tubule by passing between cells through tight junction proteins called claudins, which control which ions move through. This reabsorption is mainly driven by water, which follows sodium. Approximately 30–35% of the remaining calcium is reabsorbed in the thick ascending limb of the loop of Henle, a process mediated by intercellular proteins called claudins. Claudins also facilitate magnesium transport between cells, with their abundance increasing when magnesium is low [19]. The distal convoluted tubule and collecting ducts complete the final reabsorption of calcium via transporters such as TRPV5 on the tubular side and Na-Ca antiporters on the blood-facing side. Idiopathic hypercalciuria, the most common form, usually results from genetic changes that alter these reabsorption processes [20].

Several mechanisms have been proposed to explain the association between hypercalciuria, nephrolithiasis, and bone fragility [21]. First, increased intestinal calcium absorption, termed absorptive hypercalciuria, is often driven by upregulation of the vitamin D receptor (VDR). In specific individuals, this boosted absorption results from elevated calcitriol (1,25(OH)2D) activity and/or increased VDR expression or sensitivity. Genetic association studies implicate VDR-related pathways in the pathogenesis of absorptive hypercalciuria. Enhanced intestinal absorption increases the filtered calcium load, leading to greater urinary calcium loss if renal compensation is insufficient [22]. Second, a primary increase in bone resorption with osteoclastic activation may result from systemic biochemical changes, such as an altered parathyroid hormone (PTH) setpoint, or from intracellular signaling within bone cells. In some patients, elevated markers of bone turnover accompany urinary calcium loss, indicating that bone is the source of the released calcium. Systemic factors, including calcitriol, cytokines, and RANKL/OPG balance, as well as local osteocyte and osteoblast signaling, can promote osteoclastic activation. The concept of “bone as the buffer” suggests that when renal losses exceed intestinal absorption or when endocrine factors favor resorption, bone mineral is mobilized to maintain normal serum calcium [6,23]. Third, renal calcium wasting may result from defects in renal epithelial calcium transport, such as TRPV5/6 dysfunction or claudin abnormalities, as well as dysregulation of endocrine modulators, including FGF23/klotho and PTH. Renal distal tubular reabsorption of calcium, mediated by TRPV5 (apical entry), calbindins, and basolateral extrusion mechanisms, is essential for the reclamation of filtered calcium. Genetic and experimental disruption of TRPV5 reduces renal calcium reabsorption, leading to hypercalciuria [24]. Recent human genetic studies have shown that biallelic TRPV5 loss-of-function causes autosomal recessive renal calcium-wasting hypercalciuria, providing direct evidence linking defective renal epithelial calcium transport to systemic calcium loss and bone consequences [25,26].

Additional paracellular pathways, including claudin family proteins in the proximal nephron and thick ascending limb, and differences in sodium handling, given the close coupling of sodium and calcium transport, also influence urinary calcium excretion [27]. Polymorphisms in genes encoding proteins involved in tubular calcium reabsorption (CASR, CLDN14, TRPV6, TRPV5) or in the prevention of calcium salt precipitation (CaSR, MGP, OPN, UMOD) have been associated with idiopathic hypercalciuria and kidney stones [28]. Finally, interactions among endocrine modulators such as PTH, FGF23/Klotho, and calcitriol are critical. The endocrine axis regulates both renal and bone calcium fluxes. Elevated calcitriol increases intestinal absorption and can stimulate bone resorption. While PTH typically increases renal calcium reabsorption, chronically altered PTH dynamics, including impaired secretion set points or episodic changes, are observed in hypercalciuric populations. FGF23 and its cofactor Klotho regulate distal tubular TRPV5 abundance and, consequently, renal calcium reabsorption. Mouse models have demonstrated that FGF23 influences TRPV5 membrane expression and calcium handling. Dysregulation of these hormones can therefore increase urinary calcium excretion and affect bone remodeling [29].

Systemic disorders that are associated with both hypercalcemia and hypercalciuria include excess glucocorticoid, hyperthyroidism, hypervitaminosis D, metastatic bone tumors, paraneoplastic syndromes, milk-alkali syndrome resulting from excessive oral calcium ingestion, multiple myeloma, Paget disease, sarcoidosis, and other granulomatous disorders [6].

The identification of hypercalciuria should lead to a bone assessment, such as Dual-energy X-ray Absorptiometry (DXA), given its established association with low bone mineral density. Laboratory evaluation should include urinary calcium (24 h collection or spot urine Ca/Cr), serum calcium, parathyroid hormone (PTH), 25(OH)_2_D, and 1,25(OH)_2_D. Additional assessment of urinary lithogenic factors and bone turnover markers may be warranted in selected cases [22].

### 3.3. Metabolic Acidosis

Metabolic acidosis adversely affects bone, which functions as part of the intracellular buffer system by providing cations such as sodium, potassium, and calcium in exchange for protons, and by providing carbonate and phosphate to the extracellular fluid, consequently maintaining blood pH [30]. The acidic pH of the extracellular fluid also directly affects the transcriptome, promoting morphological changes in osteoclasts and increasing their activity [31]. In addition to these cellular effects, an acidic environment directly inhibits osteoblastic activity. It stimulates osteoclastic activity through proton-sensitive receptors, including the G protein-coupled receptor 1 of ovarian cancer (OGR1), and mediates this effect via phosphoinositide metabolites and increased PGE2 levels [30]. At the renal level, metabolic acidosis reduces tubular calcium reabsorption. For instance, metabolic acidosis can directly downregulate claudin-2 in the proximal tubule, TRPV5 and calbindin-D28K in the distal tubule, and, indirectly, inhibit claudins 16–19 in the thick ascending limb of Henle, thereby promoting hypercalciuria [24,32]. Metabolic acidosis should be recognized as a cause of secondary osteoporosis. Although most patients with metabolic acidosis have chronic kidney disease and reduced glomerular filtration rate (eGFR < 60 mL/min/1.73 m^2^), certain conditions can cause metabolic acidosis despite preserved GFR due to defects in urinary acidification. Renal tubular acidosis (RTA) illustrates this, being characterized by hyperchloremic metabolic acidosis resulting from decreased tubular capacity for urinary acidification. RTA may be hereditary or acquired through various diseases or drugs [33,34,35,36,37,38].

Renal tubular acidosis (RTA) is classified into different types: proximal RTA (pRTA or type 2), resulting from decreased proximal tubular reabsorption of filtered bicarbonate; classic distal RTA (dRTA or type 1), due to reduced proton secretion in the collecting duct; distal RTA with bicarbonaturia (type 3); distal RTA voltage-dependent with hyperkalemia (hyperkalemic type 1); and distal RTA with hyperkalemia from decreased aldosterone action in the collecting duct (type 4). pRTA (type 2) and dRTA (type 1) generally have a greater impact on bone, particularly in hereditary forms, and are usually associated with normokalaemia or hypokalaemia. In pRTA, there is often concurrent renal loss of other solutes, such as phosphorus, uric acid, glucose, and amino acids, as observed in Fanconi syndrome. In dRTA, hypercalciuria, hypocitraturia, nephrocalcinosis, and nephrolithiasis are common [35,39]. RTA should be considered in patients with suspected secondary osteoporosis, cognitive disorders, deformities from rickets or osteomalacia, hypokalemia, hypophosphatemia, nephrocalcinosis, recurrent nephrolithiasis, and especially metabolic acidosis with a disproportionately alkaline urinary pH in early-morning urine samples (urine pH > 6.0) [40].

In the outpatient setting, the primary test for diagnosing metabolic acidosis (excluding chronic respiratory disorders) is measurement of serum total CO_2_ (bicarbonate), which accounts for approximately 95% of total CO_2_. This test is widely available and can be performed using a standard biochemistry tube. A total CO_2_ value below 22 mEq/L confirms metabolic acidosis and necessitates prompt evaluation for RTA by a nephrologist. The absence of systemic metabolic acidosis characterizes an “incomplete” form of RTA. Patients with recurrent nephrolithiasis, nephrocalcinosis, hypokalemia, or other clinical and laboratory findings suggestive of RTA, even in the absence of metabolic acidosis, should be referred to a nephrologist for assessment of tubular acidification capacity using functional tests [35,41].

## 4. What Are the Indicators for Suspecting Impaired Tubular Renal Function?

Patients with bone fragility should be evaluated for renal tubulopathy if specific indicators are present. These include exposure to nephrotoxic medications (such as tenofovir, adefovir, cisplatin, ifosfamide, aminoglycosides, high-dose proton-pump inhibitors, and some chemotherapeutics); a history of cancer (especially tumor-induced osteomalacia) or monoclonal gammopathy (such as light-chain proximal tubulopathy); a family history of kidney stones, rickets or osteomalacia, salt wasting, or early fractures, which may suggest a genetic tubulopathy; and symptoms of proximal or distal tubulopathies, including polyuria or nocturia, polydipsia, bone pain, muscle weakness, recent nephrolithiasis, cardiac arrhythmias from electrolyte imbalance, and episodes of dehydration.

## 5. How to Approach Investigating Tubular Renal Function in Outpatient Settings?

A focused evaluation of tubular function combines clinical history, basic blood tests, spot and 24 h urine measurements, and selected laboratory calculations. This includes measuring fractional excretion of solutes, evaluating urinary biomarkers of tubular injury, and assessing urinary concentration and acidification capacity. The following serum and urine tests are recommended, ideally collected together:

These are the first-line laboratory tests:

(a) Serum: creatinine, electrolytes (Na, K, Ca, P, Cl, HCO_3_^−^), alkaline phosphatase, 25(OH)_2_D, iPTH.
(b) Urine single sample (spot urine): Urinalysis (dipstick), and urinary pH by potentiometry (for RTA workup).
(c) Urine single sample (spot urine): Low-molecular-weight proteins: Urinary β_2_-microglobulin, α_1_-microglobulin, or retinol-binding protein (RBP) serves as a sensitive marker of proximal tubule injury (Fanconi pattern). α_1_-microglobulin and RBP are more stable in acidic urine than β_2_-microglobulin and may therefore be preferable [42,43].
(d) Urine 24 h: creatinine, electrolytes (Na, K, Cl, Ca, P, HCO_3_^−^), glucose, uric acid, urea, citrate, protein, albumin.

After the initial laboratory results are available, multiple important derived indices should be calculated.

(a) Fractional excretion (FE) of solutes: The fractional excretion of phosphate, potassium, magnesium, uric acid, and bicarbonate supplies important diagnostic information. FE (solute) is calculated as follows:

FE (solute) = ([Solute urine × Creatinine serum]/[Solute serum × Creatinine urine]) × 100. For FE of magnesium, serum magnesium must be multiplied by 0.7 [7].

(b) TmP/GFR (tubular maximum reabsorption of phosphate per glomerular filtration rate) provides a physiologically relevant estimate of renal phosphate handling that accounts for filtered load. A low TmP/GFR indicates inappropriate renal phosphate waste relative to serum phosphate concentration. Online calculators and laboratory protocols are available. Typical ranges vary by age [44].
(c) 24 h urinary calcium (or urine Ca/Cr ratio on a spot sample): These tests detect hypercalciuria (Ca > 4 mg/Kg/24 h or Ca/Cr ratio > 0.2). Calcium excretion should be treated as a continuous variable, as values exceeding 150 mg/24 h are associated with an increased risk of nephrolithiasis [22].
(d) 24 h urinary glucose: In euglycemic patients, glucosuria over 500 mg/24 h strongly suggests proximal tubular dysfunction, such as partial or complete Fanconi syndrome.
(e) Aminoaciduria: Detection requires specialized assays, such as thin-layer chromatography, available in select tertiary laboratories [45].
(f) Acid-base testing: Assessment should include serum bicarbonate and urine pH measured by potentiometry. An inability to acidify urine in the presence of systemic acidosis suggests distal renal tubular acidosis (Type 1). Proximal RTA (Type 2), which is associated with Fanconi syndrome, presents with bicarbonaturia and, in severe cases, low serum bicarbonate. Measurement of urinary ammonium (UNH_4_^+^) further evaluates the acidification capacity of the distal nephron [35,46]. It is worth noting that the urine anion gap and osmolal gap have traditionally been used as indirect measures of UNH_4_^+^, but recent studies have shown low accuracy with this approach [47,48].

If indicated, additional specialty tests may be requested to clarify the etiology of tubulopathy:

(a) 1,25(OH)_2_D (calcitriol) and PTHrP (PTH-related peptide): both tests are indicated in cases of hypercalcemia caused by calcitriol (for instance, granulomatous diseases) or by bone, respectively [6].
(b) FGF23: This test is indicated in cases of phosphate waste without classical Fanconi features or when tumor-induced osteomalacia is suspected [10].
(c) Serum and urine electrophoresis, along with free light chain analysis, are essential for detecting monoclonal gammopathies that may cause light-chain proximal tubulopathy [49].
(d) Genetic testing: DNA analysis is essential in cases of unexplained lifelong renal phosphate or calcium waste, renal tubular acidosis, particularly when there is a positive family history, early onset of disease, or affection of multiple organs [50]. Contemporary panels for hereditary tubulopathies assess nearly one hundred genes (including *SLC34A1*, *SLC34A3*, *PHEX*, *FGF23*, *CLCN5*, *OCRL*, among others).

**Figure 1:**
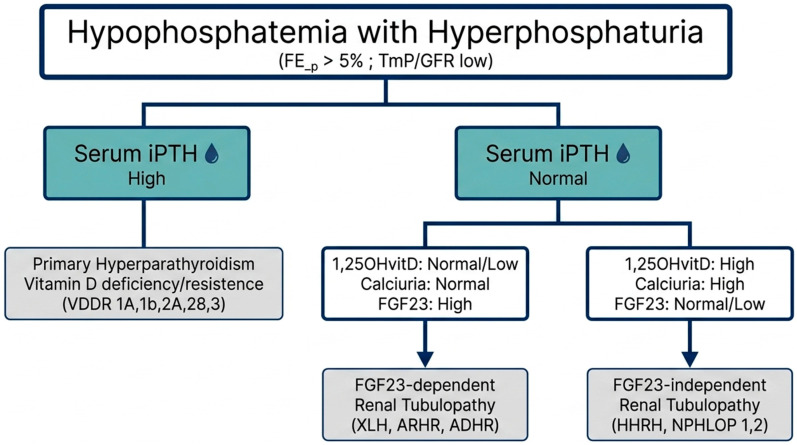
Flowchart for the diagnosis of Hyperphosphaturia. FEp: fractional excretion of phosphorus; TmP/GFR: tubular maximum reabsorption of phosphate per Glomerular filtration rate; VDDR: Vitamin D-dependent Rickets types 1A,1B,2A,2B,3; XLH: Hypophosphatemic Rickets X-linked; ARHR: AR Hypophosphatemic Rickets; ADHR: AD Hypophosphatemic Rickets; HHRH: Hypophosphatemic Rickets with Hypercalciuria; NPHLOP: Nephrolithiasis/Hypophosphatemic Osteoporosis types 1,2.

**Figure 2:**
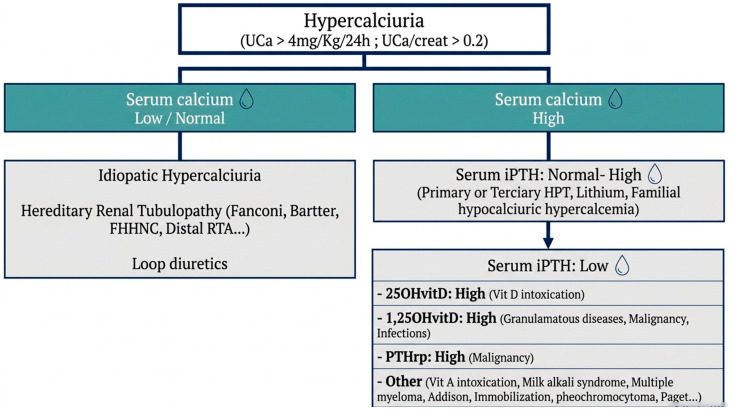
Flowchart for the diagnosis of Hypercalciuria. Uca: urinary calcium; RTA: Renal tubular acidosis; FHHNC: Familial Hypomagnesemia with Hypercalciuria and Nephrocalcinosis; HPT: hyperparathyroidism; PTHrp: Parathyroid hormone-related protein.

**Figure 3:**
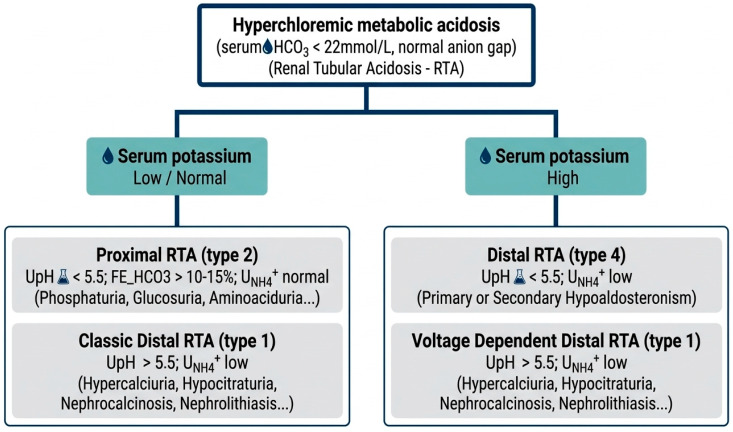
Flowchart for the diagnosis of Renal Tubular Acidosis. UpH: urinary pH; U_NH4_^+^: urinary ammonium.

Diagnostic algorithms for the principal tubulopathies associated with bone fragility, based on the predominant renal manifestation (hyperphosphaturia, hypercalciuria, or renal tubular acidosis), are provided in Figure 1, Figure 2 and Figure 3.

After diagnosing renal tubular dysfunction, it is essential to determine whether the tubulopathy is acquired or hereditary to guide treatment and prognosis. In acquired cases, treating or removing the underlying systemic disease, drug, or toxin can restore tubular function and improve bone fragility [51]. In monogenic hereditary cases, some tubulopathies already have treatments targeting the metabolic or genetic defect (enzyme replacement, specific anti-receptor antibodies, RNA interference drugs, CRISPR-Cas9-based drugs) that may cure the condition [52].

## 6. What Are the Leading Causes of Tubulopathies Associated with Bone Fragility, Including Inherited and Acquired Disorders That Disrupt Renal Tubular Function and Mineral Homeostasis?

Numerous inherited disorders of tubular transport, detailed in Table 1, Table 2, Table 3 and Table 4, are primary genetic causes of tubulopathies and often appear in childhood. However, adult-onset or late-diagnosed cases are common and may first present with fractures or low bone mineral density (BMD), as discussed below.

(a) Disorders causing renal phosphate waste include X-linked hypophosphatemia (XLH, PHEX mutations), the most significant FGF-23-mediated disorder. XLH leads to hypophosphatemia, rickets in children, and osteomalacia or fractures in adults, and is typically lifelong, sometimes recognized late [12]. Hereditary hypophosphatemic rickets with hypercalciuria (HHRH, SLC34A3/NaPi-IIc mutations) causes renal phosphate wasting, low serum phosphate, elevated 1,25(OH)_2_D, suppressed PTH, and hypercalciuria. Adults may present with bone pain and multiple fractures. HHRH is treated with phosphate replacement instead of FGF-23 antagonists [2]. Tumor-induced osteomalacia (TIO), due to FGF-23-producing mesenchymal tumors, results in severe phosphaturia, osteomalacia, and fractures; tumor localization is the primary therapeutic goal [13]. Cystinosis and other inherited proximal tubulopathies cause Fanconi syndrome with severe phosphate loss and early bone disease. Adult survivors may present with bone fragility [53].
(b) Disorders with hypercalciuria, nephrocalcinosis, and bone loss include Dent disease (CLCN5 or OCRL mutations), an X-linked tubulopathy with low-molecular-weight proteinuria, hypercalciuria, nephrolithiasis or nephrocalcinosis, and progressive CKD. Bone consequences, such as BMD reduction and fractures, are increasingly recognized. Early recognition prevents nephrocalcinosis and bone complications [53]. Bartter syndrome variably affects bone, with some forms causing impaired growth and bone mass [6].

The leading causes of acquired tubulopathies resulting in bone fragility (Table 5) include:

(a) Drug-induced proximal tubulopathy and Fanconi syndrome: Tenofovir disoproxil fumarate (TDF) is a well-documented cause of proximal tubular dysfunction, Fanconi syndrome, hypophosphatemic osteomalacia, and fracture. Risk factors include older age, low body weight, coadministration of boosters, preexisting kidney disease, and prolonged exposure. Clinicians should suspect tenofovir-related tubulopathy in HIV/HBV patients with bone pain, hypophosphatemia, or proximal muscle weakness. Tenofovir alafenamide (TAF) causes less tubulopathy but still needs vigilance [54]. Ifosfamide is a chemotherapeutic agent known to cause delayed proximal tubular dysfunction and osteomalacia, mainly in children but also in adults post-chemotherapy, with insidious and late tubular damage. Platinum-based chemotherapy, aminoglycosides, contrast agents, and heavy metals may cause tubular injury; persistent proximal injury can lead to phosphate waste and bone effects [49]. Adefovir and other nucleotide antivirals are also associated with proximal tubular injury and phosphate waste [49].
(b) Systemic diseases causing acquired tubulopathies: Multiple myeloma and monoclonal light-chain–associated proximal tubulopathy commonly present as Fanconi-type syndrome with osteomalacia and fragility fractures. Screening with serum/urine protein electrophoresis and free light chains is essential. Autoimmune diseases, such as Sjögren’s syndrome, cause distal RTA and osteomalacia; other tubulointerstitial nephritides (e.g., sarcoidosis) may affect tubular mineral handling [36].
(c) Environmental toxins: Heavy metals like lead and cadmium, and other exposures, can cause tubular dysfunction and mineralization defects. Chronic exposure should be identified with a thorough patient history and investigated if suspected [49].

## 7. How Should Patients with Tubulopathies Associated with Bone Disease Be Treated, with a Focus on Correcting Electrolyte and Acid-Base Disturbances and Targeting the Underlying Cause?

Essential steps include removing offending drugs or toxins. Drug withdrawal may improve tubular markers and bone pain [55]. Genetic counseling and multidisciplinary care are recommended [12].

Conventional therapy for many hypophosphatemic disorders includes oral phosphate in divided doses and active vitamin D analogs (calcitriol or alfacalcidol) to promote mineralization. This calls for close monitoring to prevent secondary hyperparathyroidism and nephrocalcinosis, and for close surveillance of urinary calcium to avoid hypercalciuria [5]. Management may involve treating the underlying cause and repairing mineralization. For instance, surgical resection of FGF23-secreting tumors cures TIO, while genetic causes require long-term management. Monoclonal antibodies against FGF23, like burosumab, have been developed for XLH and FGF23-mediated hypophosphatemia, improving phosphate, 1,25(OH)_2_D, and skeletal outcomes [56]. This approach shows that understanding phosphate regulation enables the development of disease-modifying treatments [5].

In hypercalciuria, thiazide diuretics reduce urinary calcium by enhancing tubular calcium reabsorption, therefore improving urinary calcium excretion and, in some studies, BMD [23,38]. It is noteworthy that a recent randomized controlled clinical trial showed that indapamide had potential bone-protective effects, substantially reducing β-crosslaps compared with hydrochlorothiazide [57]. Dietary priorities include moderate sodium restriction, adequate calcium intake, and sufficient vitamin D. Low calcium intake is not recommended, as it may increase the risk of stone formation and bone loss. Vitamin D supplementation should be personalized, given the variation in stone risk [58]. In patients with osteoporosis and hypercalciuria, antiresorptive drugs (bisphosphonates, denosumab) may increase BMD; correcting hypercalciuria first may enhance antiresorptive effects [17]. Bone-directed antiresorptive drugs must be administered cautiously, only after excluding osteomalacia or treating it, as potent antiresorptive drugs are contraindicated in untreated osteomalacia. Correction of phosphate and vitamin D levels, as well as therapy of tubulopathy, must precede the use of antiresorptive drugs [5].

When treating metabolic acidosis due to renal tubular acidosis (RTA), it is essential to identify the underlying cause to guide a specific approach, such as in Sjögren’s syndrome in adults. Oral alkaline therapy—using potassium citrate, sodium bicarbonate, or SHOHL solution—is the primary treatment. Calcitriol may be used for proximal RTA (pRTA), along with other steps to support bone health [39]. When initiating osteoporosis medications, note that RTA can also lead to reduced bone remodeling, including adynamic bone or osteomalacia [30]. Because of this, as mentioned before, antiresorptive drugs such as bisphosphonates and anti-RANKL therapies ought to be administered cautiously, ideally after assessing bone remodeling with a tetracycline-labeled bone biopsy or after an anabolic agent has been used [59].

For long-term monitoring of patients with bone diseases caused by renal tubular disorders, it is important to routinely assess serum phosphate, calcium, and bicarbonate, and to evaluate urinary phosphate and calcium; if possible, also test for low-molecular-weight proteinuria. Some recent observational studies, for example, suggest an association between abnormal serum levels of uric acid and aldosterone and an increased risk of osteoporosis and fractures in middle-aged and elderly patients with hypertension [60,61]; therefore, long-term monitoring is also recommended in cases of tubulopathies involving abnormalities in these electrolytes or hormones.

After correcting all mineral imbalances, serial DXA bone scanning, including the trabecular bone score (TBS) when possible, is also recommended to monitor changes in bone mineral density in these patients [62,63].

## 8. Limitations

Several limitations are inherent to this review. As a narrative review, it remains susceptible to selection and publication bias, even with a structured literature search. The included studies varied in design, study type (prognostic, diagnostic, or treatment), and patient populations [64,65,66,67,68,69,70,71,72,73]. Furthermore, many studies were retrospective, conducted at single centers, or involved small sample sizes, which limits the generalizability of the findings [74,75,76,77,78,79,80,81,82]. Future research should prioritize randomized clinical trials, as they remain underrepresented in studies of renal tubulopathies.

## 9. Conclusions

Renal tubular disorders are a significant and often under-recognized cause of bone hypomineralization and fragility fractures in adults. Accurate diagnosis requires a systematic approach that includes clinical history; targeted laboratory testing (serum calcium, phosphate, and bicarbonate); calculated indices (fractional excretion of phosphate, tubular maximum reabsorption of phosphate per glomerular filtration rate, urinary calcium/creatinine ratio); urine pH; and low-molecular-weight protein assays. These steps help identify various tubulopathies, such as drug-induced Fanconi syndrome, genetic phosphate-wasting disorders, hypercalciuric states, and renal tubular acidosis. Early recognition and intervention, including withdrawal of nephrotoxic drugs, phosphate and vitamin D supplementation, alkali therapy, thiazide diuretics, tumor localization and removal, and disease-specific therapies, are important for preventing further fractures and renal injury. Coordinated multidisciplinary care involving nephrologists, endocrinologists, rheumatologists, metabolic bone disease specialists, and geneticists is often required for complete evaluation and management.

## 10. Practical Clinical Pearls

In adults with multiple low-impact fractures and bone pain, clinicians should consider causes beyond osteoporosis. Assessment of possible renal tubulopathy should include serum and urine phosphate, serum and urine calcium, serum bicarbonate, and urinalysis before starting antiresorptive therapy.

Simultaneous collection of serum and urine samples is necessary for calculating the tubular maximum reabsorption of phosphate per glomerular filtration rate and the fractional excretion of all electrolytes.

Low-molecular-weight proteinuria testing (β_2_-microglobulin, α_1_-microglobulin, or retinol-binding protein) is a sensitive early marker of proximal tubular injury. It should be performed when a suspected acquired or inherited renal tubulopathy is identified.

Hereditary renal tubulopathy may be presented in adulthood with fractures or nephrolithiasis. Clinicians should have a low threshold for genetic referral if the phenotype is suggestive.

## Figures and Tables

**Table 1:** Inherited renal tubular disorders causing Fanconi syndrome.

Disease	G.I.	Gene Protein	Main Clinical Characteristics
Lowe Syndrome	XR	*OCRL* OCRL1	Blindness, rickets, neuropsychomotor deficit, facial dysmorphia
Dent Syndrome (1, 2)	XR	*CLCN5/OCRL* CLC5/OCRL1	Rickets, hypercalciuria, nephrocalcinosis, nephrolithiasis
Cystinosis	AR	*CTNS* Cistinosin	Impaired growth, hepatomegaly hypophosphatemic rickets
Fanconi-Bickel Syndrome	AR	*SLC2A2* GLUT2	Impaired growth, hepatomegaly hypophosphatemic rickets, hypoglycemia
Hartnup Disease	AR	*SLC6A19* B(0)AT1	Cerebellar ataxia, malabsorption, “pellagra-like,” malnutrition
Lysinuric Protein Intolerance (LPI)	AR	*SLC7A7* γLAT1	Malnutrition, hypotonia, hepatosplenomegaly
Wilson Disease	AR	*ATP7B* Cu-P-type ATPase	Kayser-Fleischer rings, liver cirrhosis, CNS disorders
Tyrosinemia	AR	*FAH/TAT/HPD* Specific enzymes	Impaired growth, hepatomegaly hypophosphatemic rickets
Galactosemia (types I, II, III)	AR	*GALT/GALK/GALE* GALT/GALK/GALE	Cataracts, liver disease, encephalopathy, TGI disorders
Fructose Intolerance	AR	*Aldo B* Aldolase B	Hypoglycemia, vomiting, liver disease
Fanconi Renotubular Syndrome (FRTS1)	AD	*GATM* (others) Gly Amidinotransferase	Impaired growth, polyuria, acidosis, hypophosphatemic rickets
Fanconi Renotubular Syndrome (FRTS2)	AD	*SLC34A1* (others) NaPi2a	Impaired growth, polyuria, hypercalciuria, acidosis, hypophosphatemic rickets
Fanconi Renotubular Syndrome (FRTS3)	AD	*EHHADH* EHHADH	Impaired growth, polyuria, acidosis, hypophosphatemic rickets
Fanconi Renotubular Syndrome (FRTS4)	AD	*HNF4A* HNF4αptn	Impaired growth, MODY 1, macrosomia, nephrocalcinosis
Fanconi Renotubular Syndrome (FRTS5)	AR	*NDUFAF6* (others) NDUFAF6	Pulmonary fibrosis, polyuria, acidosis, hypophosphatemic rickets
Imerslund-Gräsbek Syndrome	AR	*CUBN/AMN* Cubilin/Amnionless	CNS disorders, hypotony, hypovitaminosis B12
Donnai-Barrow Syndrome	AR	*LRP2* Megalin	Diaphragmatic hernia-omphalocele, agenesis of the corpus callosum
ARC Syndrome (Arthrogryposis/Renal/Cholestasis)	AR	*VPS33B/VIPAR* Apical proteins	Cholestasis, ichthyosis, thrombocytopenia, CNS disorder, osteoarthritis

G.I.—Genetic inheritance; AD—Autosomal dominant; AR—Autosomal recessive; XR—X-linked.

**Table 2:** Inherited renal tubular disorders causing hyperphosphaturia.

Disease	G.I.	Gene Protein	Serum P	Serum Ca	Serum 1,25OHD	Serum iPTH	Serum FGF23
Hypophosphatemic Rickets X-linked (XLH)	XR	*PHEX* Endopeptidase	Low	Normal	Low/Normal	High/Normal	High
Hypophosphatemic Rickets AR (ARHR types 1,2)	AR	*DMP1/ENPP1* DMP1/PPi	Low	Normal	Low/Normal	High/Normal	High/Normal
Hypophosphatemic Rickets AD (ADHR)	AD	*FGF23* FGF23	Low	Normal	Low/Normal	High/Normal	High
Hypophosphatemic Rickets With Hyperparathyroidism	?	*KLOTHO* KLOTHO	Low	High/Normal	Low/Normal	High	High
Hypophosphatemic Rickets With Hypercalciuria (HHRH)	AR	*SLC34A3* NaPi2c	Low	Normal	High	Low	Low/Normal
Nephrolithiasis/Hypophosphatemic Osteoporosis 1 (NPHLOP1)	AD	*SLC34A1* NaPi2a	Low	High/Normal	High	Normal	Low/Normal
Nephrolithiasis/Hypophosphatemic Osteoporosis 2 (NPHLOP2)	AD	*SLC9A3R1* NHERF1	Low	Normal	High	Normal	Low
Vitamin D-dependent Rickets 1A (VDDR-1A)	AR	*CYP27B1* 1α-hidroxilase	Low	Low	Low	High	Low/Normal
Vitamin D-dependent Rickets 1B (VDDR-1B)	AR	*CYP2R1* 25-hidroxilase	Low	Low	Low/Normal	High	Low/Normal
Vitamin D-dependent Rickets 2A (VDDR-2A)	AR	*VDR* VDR	Low	Low	High	High	Low/Normal
Vitamin D-dependent Rickets 2B (VDDR-2B)	AR	*HNRNPC* VDR	Low	Low	High	High	Low/Normal
Vitamin D-dependent Rickets 3 (VDDR-3)	AR	*CYP3A4* CYP3A4	Low	Low	Low	High	?

G.I.—Genetic inheritance; AD—Autosomal dominant; AR—Autosomal recessive; XR—X-linked.

**Table 3:** Inherited renal tubular disorders causing hypercalciuria.

Disease	G.I.	Gene Protein	BP	Serum HCO_3_	Serum K	Serum Mg	Serum Ca	Serum P	Urine Mg	Urine P
Bartter Syndrome type 1	AR	*SLC12A1* NKCC2	Low/N	High	Low	Low /N	N	N	High /N	N
Bartter Syndrome type 2	AR	*KCNJ1* ROMK	Low/N	High	Low	Low /N	N	N	High /N	N
Bartter Syndrome type 3	AR	*CLCNKB* ClC-Kb	Low/N	High	Low	Low	N	N	High	N
Bartter Syndrome type 4a/4b (digenic)	AR	*CLCNKA-CLCNKB* Barttin	Low/N	High	Low	Low /N	N	N	High /N	N
Bartter Syndrome type 5	XR	*MAGED2* MAGED2	Low/N	High	Low	?	Low /N	N	?	N
Familial Hypomagnesemia with Hypercalciuria and Nephrocalcinosis (FHHNC)	AR	*CLDN16/* *CLDN19* Claudins 16/19	Low/N	N	Low	Low	Low /N	N	High	N
Autosomal Dominant Hypocalcemia	AD	*CASR* CaSR (Gain)	N	N	N	N	Low	N	N	N
Pseudohypoparathyroidism Ia (Albright’s Hereditary Osteodystrophy)	AD	*GNAS* Prot Gsα	N	N	N	N	Low	High	N	Low
Hereditary Absorptive Hypercalciuria?	?	*ADCY10* Adenylyl cyclase	N	N	N	N	N	N	N	N

G.I.—Genetic inheritance; BP—Blood pressure; AD—Autosomal dominant; AR—Autosomal recessive; XR—X-linked; N—normal.

**Table 4:** Inherited renal tubular disorders causing renal tubular acidosis (RTA).

Disease	G.I.	Gene Protein	Serum HCO_3_	Serum K	Urine HCO_3_	Urine pH	Urine Ca
Proximal RTA (type 2)	AR	*SLC4A4* NBC1	Low	Low/Normal	High	pH < 5.5	Normal
Proximal RTA (type 2) transient	AD	*SLC9A3* NHE-3	Low	Low/Normal	High	pH < 5.5	Normal
Proximal + Distal RTA (type 3)	AR	*CA2* CA2	Low	Low/Normal	High	pH > 5.5	Normal
Distal RTA (type 1)	AD	*SLC4A1* AE1	Low	Low/Normal	Normal	pH > 5.5	High
Distal RTA (type 1) with hemolytic anemia	AR	*SLC4A1* AE1	Low	Low/Normal	Normal	pH > 5.5	High
Distal RTA (type 1) with hearing loss	AR	*ATP6V1B1* VATPaseB1	Low	Low/Normal	Normal	pH > 5.5	High
Distal RTA (type 1)	AR	*ATP6V0A4* VATPaseBa4	Low	Low/Normal	Normal	pH > 5.5	High
Distal RTA (type 1) with hearing loss	AR	*FOXI1* Forkhead ptn	Low	Low/Normal	High	pH > 5.5	High
Distal RTA (type 1)	AR	*WDR72* WDR protein 72	Low	Low/Normal	High	pH > 5.5	High
Distal RTA (type 4) Pseudohypoaldosteronism type 1a	AD	*MLR* MR	Low	High	Normal	pH < 5.5	Normal
Distal RTA (type 4) Pseudohypoaldosteronism type 1b	AR	*SCNN1* ENaC	Low	High	Normal	pH < 5.5	Normal
Distal RTA (type 4) Gordon Pseudohypoaldosteronism type 2	AD	*WNK 1,4* WNK proteins	Low	High	Normal	pH < 5.5	High

G.I.—Genetic inheritance; AD—Autosomal dominant; AR—Autosomal recessive.

**Table 5:** Acquired renal tubular disorders and nephron involvement.

Systemic Conditions	Proximal Nephron	Distal Nephron	Drugs/Toxins	Proximal Nephron	Distal Nephron
Multiple Myeloma/Amyloidosis (AL)	++++	++	Heavy metals	++++	++
MGRS (Monoclonal Gammopathy of Renal Significance)	++++	++	Ifosfamide	++++	+
Chronic Lymphocytic Leukemia	+	+	Tenofovir (TDF)	++++	+
Waldenström macroglobulinemia	+	+	Aminoglycosides	++++	+
Hyperparathyroidism	++	++	Cisplatin/Oxaliplatin	++++	++
Sarcoidosis	++	++	Acetazolamide/Topiramate	++++	++
Post kidney transplant	++++	++++	Hypervitaminosis D	++	++
Alport Syndrome	+	+	Lithium	++	++++
Sjogren Syndrome	++	++++	Amphotericin B	+	++++
Systemic Lupus Erythematosus	+	+	Trimethoprim	-	++
Rheumatoid Arthritis	+	+	Amiloride	-	++
Mitochondrial Cytopathies	++++	++	Toluene	++	++++
Hashimoto/Graves	+	+	Pentamidine	-	++
Adrenal Insufficiency	+	++	Heparin	-	++
Primary biliary cholangitis/Cirrhosis	+	+	Spironolactone	-	++
Autoimmune Hepatitis	+	+	ACEs/ARBs	++	++
Primary Immunodeficiency	++	++	NSAIDS	++++	++++
HIV Infection	++	++	Cyclosporin/Tacrolimus	++	++++
HCV-related cryoglobulinemia	+	+	Rapamycin	++	++
Sickle Cell Disease	++	++++	Succinylcholine	+	+
Nephrocalcinosis	++	++++	Checkpoint Inhibitors	++	++
Obstructive Uropathy	+	++++	TK inhibitors	++	++
Vesicoureteral reflux	+	++++	VEGF inhibitors	++	++
Medullary Sponge Kidney	+	++++	Proteasome inhibitors	++	++
Diabetes Renal Disease	++	+++	Lena/Pomalidomide	++	++
Chronic Tubulointerstitial Nephritis	++++	++++	EGFR inhibitors	++	++
Transthyretin amyloidosis (ATTR)	++	++	Iodinated contrasts	++++	++

TDF—Tenofovir disoproxil fumarate; ACE—Angiotensin-converting enzyme; ARBs—Angiotensin II receptor-blockers; NSAIDS—Nonsteroidal Anti-inflammatory Drugs; TK—Tyrosine Kinase; VEGF—Vascular endothelial growth factor; EGFR—Epidermal growth factor; “+”—it affects the nephron segment; “-”—it does not affect the nephron segment.

## Data Availability

No new data were created or analyzed in this study. Data sharing is not applicable to this article.

## Abbreviations

The following abbreviations are used in this manuscript:

1,25(OH)_2_D	Calcitriol
25(OH)_2_D	Vitamin D3
BMD	Bone Mineral Density
CKD	Chronic Kidney Disease
dRTA	Distal Renal Tubular Acidosis
DXA	Dual-energy X-ray Absorptiometry
eGFR	Glomerular Filtration Rate
FGF23	Fibroblast Growth Factor 23
pRTA	Proximal Renal Tubular Acidosis
PTH	Parathyroid hormone
UNH_4_^+^	Urinary ammonium
